# The role of cervical collars and verbal instructions in minimising spinal movement during self-extrication following a motor vehicle collision - a biomechanical study using healthy volunteers

**DOI:** 10.1186/s13049-021-00919-w

**Published:** 2021-07-31

**Authors:** Tim Nutbeam, Rob Fenwick, Barbara May, Willem Stassen, Jason E. Smith, Lee Wallis, Mike Dayson, James Shippen

**Affiliations:** 1 Emergency Department, University Hospitals Plymouth NHS Trust, Plymouth, UK; 2 Devon Air Ambulance Trust, Exeter, UK; 3 Division of Emergency Medicine, University of Cape Town, Cape Town, South Africa; 4 University Hospitals Birmingham, Birmingham, UK; 5 Institute for Future Transport and Cities, University of Coventry, Coventry, UK; 6 Academic Department of Military Emergency Medicine, Royal Centre for Defence Medicine, Birmingham, UK; 7 Fire Officer, National Fire Chiefs Council, Birmingham, UK

## Abstract

**Background:**

Motor vehicle collisions account for 1.3 million deaths and 50 million serious injuries worldwide each year. However, the majority of people involved in such incidents are uninjured or have injuries which do not prevent them exiting the vehicle. Self-extrication is the process by which a casualty is instructed to leave their vehicle and completes this with minimal or no assistance. Self-extrication may offer a number of patient and system-wide benefits. The efficacy of routine cervical collar application for this group is unclear and previous studies have demonstrated inconsistent results. It is unknown whether scripted instructions given to casualties on how to exit the vehicle would offer any additional utility.

The aim of this study was to evaluate the effect of cervical collars and instructions on spinal movements during self-extrication from a vehicle, using novel motion tracking technology.

**Methods:**

Biomechanical data on extrications were collected using Inertial Measurement Units on 10 healthy volunteers. The different extrication types examined were: i) No instructions and no cervical collar, ii) No instructions, with cervical collar, iii) With instructions and no collar, and iv) With instructions and with collar. Measurements were recorded at the cervical and lumbar spine, and in the anteroposterior (AP) and lateral (LAT) planes. Total movement, mean, standard deviation and confidence intervals are reported for each extrication type.

**Results:**

Data were recorded for 392 extrications. The smallest cervical spine movements were recorded when a collar was applied and no instructions were given: mean 6.9 mm AP and 4.4 mm LAT. This also produced the smallest movements at the lumbar spine with a mean of 122 mm AP and 72.5 mm LAT.

The largest overall movements were seen in the cervical spine AP when no instructions and no collar were used (28.3 mm). For cervical spine lateral movements, no collar but with instructions produced the greatest movement (18.5 mm). For the lumbar spine, the greatest movement was recorded when instructions were given and no collar was used (153.5 mm AP, 101.1 mm LAT).

**Conclusions:**

Across all participants, the most frequently occurring extrication method associated with the least movement was no instructions, with a cervical collar in situ.

## Background

Motor vehicle related trauma is common – accounting for 1.3 million deaths and 50 million serious injuries per year worldwide [1]. The United Nations (UN) Sustainable Development Goals include a target to halve all road deaths and injuries by 2020 [2]. Following a Motor Vehicle Collision (MVC) up to 40% of casualties will be trapped and require extrication - these casualties have an excess morbidity and mortality [3–11].

A small proportion of casualties will remain in their vehicle following an MVC as they require disentanglement from the wreckage (physical entrapment) by rescue services [12]. These extrications require the use of cutting and spreading tools. The use of such tools may cause considerable additional vehicular damage, has significant resource implications (both human and equipment), is physically demanding and additionally subjects casualties and rescuers to a real risk of harm [13].

Other casualties may not be able to leave their vehicle due to the severity of the injuries that they have sustained. In trapped casualties with major trauma, chest injuries are the most common severe injury (abbreviated injury scale > = 3) followed by limb and then head injuries. Unstable spinal injury or spinal cord injury are infrequent [11].

Most people involved in MVCs will be uninjured or have injuries which do not prevent them exiting the vehicle. There will also be cases where those with significant injuries may be able to exit the car without formal extrication by rescue services [12].

Self-extrication is the process by which a casualty leaves their vehicle (with or without instructions) and completes this with minimal or no assistance from the rescue services [14]. Self-extrication is currently recommended by the Faculty of Prehospital Care of the Royal College of Surgeons of Edinburgh and is featured in United Kingdom (UK) Fire and Rescue Services (FRS) national guidance for performing rescues [15]. Despite having featured in this guidance since 2017, translation into practice is low, with only 3% of FRS in the UK using self-extrication on a regular basis [16].

Self-extrication is significantly quicker than tool extrication methods. Previous work has identified a mean extrication time of 30 min for tool extrication [17], whereas self-extrication can normally be completed in less than 60 s. While committed to an extrication incident, both the rescue services and the medical response that has attended are not available to respond to other requests for assistance. The time saved both on-scene and in deployment therefore has the potential to relieve some of the increasing service pressures faced by operational staff.

Fire and Rescue service guidance indicates that even minimal movement of the spinal column during extrication may be disastrous for casualties, by significantly exacerbating a spinal injury: “*with an unstable fracture, displacement of as little as one millimetre may be enough to compress, pinch or shear the spinal cord. This damage may make the difference between normal function and permanent paralysis, therefore it is imperative that no further motion occurs in an unstable spine …*” [18]. Guidance also indicates that spinal injury should be assumed to have occurred in the vast majority of MVCs: “*the presence of spinal injury must be assumed with any sudden acceleration or deceleration accident”* [18].

The role of cervical collars, particularly in conscious trauma casualties, is being increasingly questioned, with prehospital care practice moving away from the utilisation of collars in all but special circumstances (e.g. to allow facial packing in maxillofacial injury) [19–22]. These conflicting analyses suggest that the optimal role of cervical collars as an extrication device remains unclear, particularly in the setting of self-extrication [23–25].

The aim of this study is to evaluate the role of both cervical collars and instructions, in relation to cervical and lumbar spinal movements, for casualties undertaking self-extrication from a vehicle, by using motion tracking technology.

## Methods

This study is a biomechanical analysis using healthy volunteers, comparing cervical and lumbar spine movement during four types of self-extrication. The extrication types are: i) No instructions and no cervical collar, ii) No instructions, with cervical collar, iii) With instructions and no collar, and iv) With instructions and with collar.

Participants: Ten healthy volunteers were recruited to participate in this study from participating FRS centre support roles. Participants had no previous knowledge of extrication, had no back or neck conditions that may be exacerbated by extrication and had a body mass of less than 100 kg. Participants were briefed on the study, had access to a participant information sheet in advance and completed written informed consent prior to participation.

Data collection: Each participant’s height and weight were recorded prior to being fitted with the Inertial Measurement Unit (IMU) (Xsens Awinda). IMU’s are biomechanical analysis devices which include three orthogonal linear accelerometers, three orthogonal rate gyroscopes and three orthogonal magnetometers. By attaching inertial measurement unit (IMU) sensors to each of the major segments of the body, the posture can be measured and, together with a foot contact model and biomechanical model, the positioning of the subject can be recorded [26]. The accuracy of IMU based kinematic and kinetic measurements have been shown to be comparable with optical tracking methods, and has been validated for such applications therefore enabling their utilisation within clinical analysis [27, 28]. In this case, the IMU sensor was attached to the head using a headband. The thorax was assumed to be rigid and sensors were positioned over the clavicle notch on the sternum and over each scapula using a tight-fitting elastic vest. A sensor was positioned on the sacrum by attaching the sensor to shorts using hook-and-loop fastening, to prevent upward travel, and securing the sensor against the body with an elastic belt. Orientation data were collected from each sensor via a wi-fi link and sampled at a rate of 60 Hz.

Where collars were required, Laerdal Stiff Neck collars were used, and these were fitted by a member of the study team trained in their use in accordance with manufacturer guidance. The verbal instructions for extrication were taken from the work of Dixon et al. and can be found in Table 1; these instructions were delivered by a trained member of the study team [23].

**Table 1:** Instructions for Self-Extrication

Step 1 ‘Do you understand what we are asking you to do?’ Try and keep your head as still as possible. Stop at any time if you feel pain or strange sensations in your body.	
Step 2 Slowly move your right foot and place it on the ground outside the car.	
Step 3 Using the steering wheel for support pull yourself forward.	
Step 4 Keep your left hand on the steering wheel and place your right hand on the edge of the seat behind you.	
Step 5 Turn slowly on your seat to face the outside, your left leg should follow when ready but remain seated.	
Step 6 With both feet flat on the floor stand straight up using your arms for balance.	
Step 7 Take two steps away from the car.	

A power calculation was performed to determine the sample size required for this study. The existing literature in this and related fields was searched to identify a suitable minimally clinical important difference (MCID) for spinal movement in the context of prevention / minimisation of secondary injury. A MRI study reported a mean difference of 2.7 mm between spinal canal space in patients with and without cord injury in the context of bony spinal injury [29]. Despite the significant limitations of how this value was derived, previous studies of extrication recommend using this value as the MCID to power biomechanical trials of extrication [30]. This trial was powered using means and standard deviations derived from pilot data collected by this study group. The power calculation was based on finding an anterior-posterior translational movement at the cervical spine of 2.7 mm with a significance level of 1% and a power of 90%, giving a sample size of 47 per group.

Each of the ten participants repeated each of the four types of self-extrication 10 times giving a total of 100 extrications for each type and 400 extrications across the study. Data were excluded from analysis if a sensor became dislodged or data capture failed.

The vehicle type was pre-specified as a 5-door hatchback (2018 Nissan Leaf), the commonest vehicle type on UK roads [31] .

**Fig. 1:**
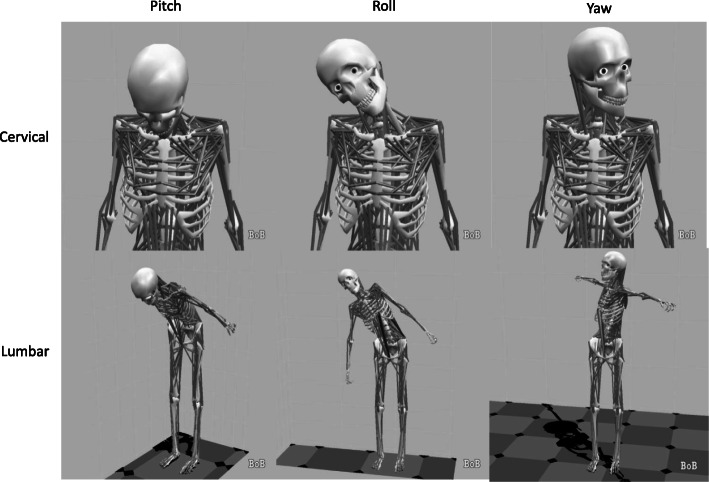
Diagrammatic representation of pitch, roll and yaw and the cervical and lumbar spine

The IMU directly measures the segmental orientations from which relative motions can be calculated and reported by assuming the relative rotations of adjacent vertebrae across the lumbar and cervical region are constant. Maximum excursions (movement from a hypothetical midline) were calculated for anterior/ posterior (AP) movement of the cervical spine and lumbar spine, and lateral (Lat) movement of the cervical spine and lumbar spine (Fig. 1).

Data were captured and analysed using the Biomechanics of Bodies (BoB, Bromsgrove, UK) software interface [32] before being exported to Excel (Microsoft v. 16.9) and SPSS (IBM v. 25, Armonk NY) for further analysis and reporting. Total excursions, standard deviation and confidence intervals are reported for each extrication type. *P* values were calculated using a two tailed t-test comparing each extrication method with Dixon’s standard (self-extrication with instructions and no collar).

The study protocol was reviewed and approved by the University of Coventry Research Ethics Committee (reference number P88416).

## Results

Data from a total of 392 extrications were successfully collected for analysis (98% data capture success rate). Seven of the ten participants were female, with a mean age across all of the participants of 39 years (range 21–59) and BMI of 25.1 (range 19–29).

**Fig. 2:**
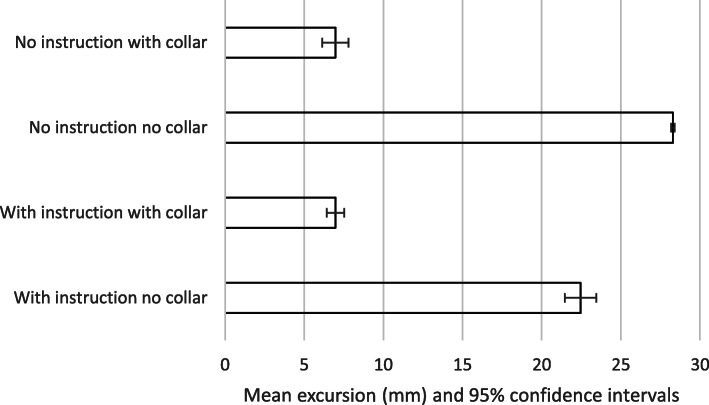
Mean excursion and confidence intervals for anterior-posterior movement at the cervical spine

**Fig. 3:**
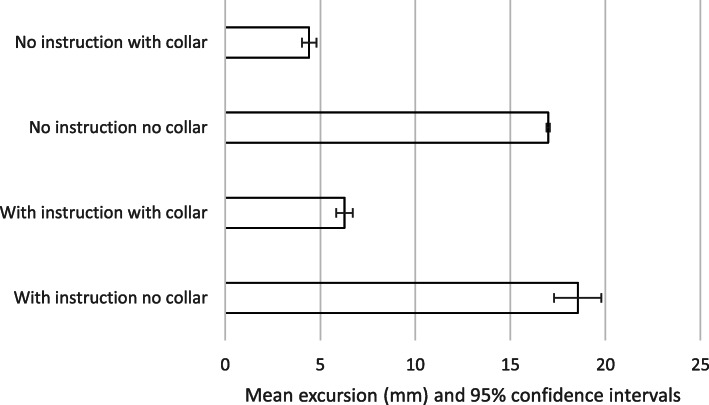
Mean excursion and confidence intervals for lateral movement at the cervical spine

**Fig. 4:**
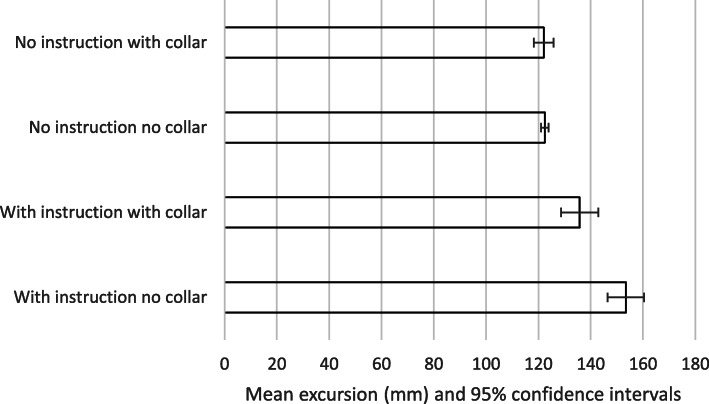
Mean excursion and confidence intervals for anterior-posterior movement at the lumbar spine

**Fig. 5:**
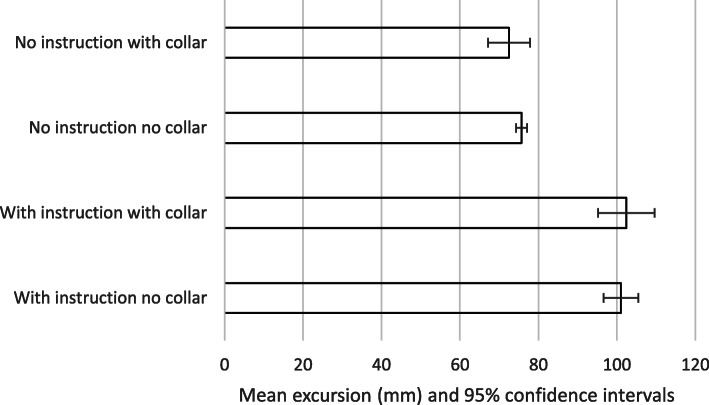
Mean excursion and confidence intervals for lateral movement at the lumbar spine

The results are summarised in Tables 2-3 and Figs. 2, 3, 4 and 5. The mean movement across the four extrication types was 16.2 mm (Cervical AP), 11.5 mm (Cervical Lat), 133.4 mm (Lumbar AP) and 87.9 mm (Lumbar Lat). Cervical roll was 21.0^o^, cervical pitch 29.9^o^ and cervical yaw 32.1^o^. Lumbar roll was 32.7^o^, lumbar pitch 42.7^o^ and lumbar yaw 40.4^o^.

**Table 2:** Participant demographics, extrications and mean AP movement

Participant	Age	Weight (kg)	Height (cm)	BMI	Sex	Extrications suitable for analysis	Mean AP cervical movement mm (SD)	
1	59	85	175	27.8	M	39	22.8	(2.6)
2	27	55	163	20.7	F	39	25.2	(1.9)
3	39	74	168	26.2	F	39	26.0	(2.7)
4	28	55	167	19.7	F	40	22.2	(7.00)
5	52	84	180	25.9	M	41	17.8	(2.2)
6	38	59	157	23.9	F	39	23.9	(2.2)
7	45	79	180	24.4	M	37	30.0	(3.7)
8	53	68	153	29.0	F	38	21.3	(2.2)
9	28	56	152	24.2	F	40	16.8	(2.6)
10	21	77	163	29.0	F	40	18.5	(3.2)
MEAN:	39.0	69.2	165.8	25.1	M:F, 3:7	Total: 392	MEAN 22.5	(5.1)

**Table 3:** Means, standard deviations and *p* values

	With instruction no collar		With instruction with collar			No instruction no collar			No instruction with collar		
	MEAN	STDEV	MEAN	STDEV	Significance (p)	MEAN	STDEV	Significance (p)	MEAN	STDEV	Significance (p)
Cervical A/P [mm]	22.5	5.1	***7.0***	***2.7***	***< 0.001***	28.3	6.9	< 0.001	***7.0***	***4.2***	***< 0.001***
Cervical Lat [mm]	18.5	6.3	6.3	2.1	< 0.001	17.0	4.6	0.02	***4.4***	***1.9***	***< 0.001***
Cervical roll [^O^]	33.9	13.0	10.8	4.0	0.2	33.3	17.5	0.36	*9.8*	*8.4*	*0.15*
Cervical pitch [^O^]	42.7	9.2	13.2	5.3	< 0.001	50.5	13.4	< 0.001	***13.0***	***7.3***	***< 0.001***
Cervical yaw [^O^]	49.3	20.3	15.0	9.0	< 0.001	54.6	19.3	0.061	***9.5***	***5.0***	***< 0.001***
Lumbar A/P [mm]	153.5	35.2	135.8	35.3	< 0.001	122.4	27.7	< 0.001	***122.0***	***19.1***	***< 0.001***
Lumbar Lat [mm]	101.1	22.5	102.5	35.4	0.54	75.7	28.0	< 0.001	***72.5***	***27.4***	***< 0.001***
Lumbar roll [^O^]	33.0	6.4	36.3	13.3	0.012	***29.0***	***10.2***	***0.001***	32.3	13.0	0.64
Lumbar pitch [^O^]	49.1	9.6	43.0	10.9	< 0.001	39.7	9.3	< 0.001	***39.1***	***7.7***	***< 0.001***
Lumbar yaw [^O^]	46.9	11.5	50.0	17.2	0.46	***31.0***	***7.8***	***< 0.001***	**33.7**	**10.9**	***< 0.001***

***Bold italics = extrication values with statistically significant smallest movement***

For the cervical spine, the smallest overall movements were recorded when a collar was applied and no instructions were given (6.9 mm AP and 4.4 mm LAT). These were also the conditions producing the smallest movements at the lumbar spine (122 mm AP and 72.5 mm LAT).

The largest overall movements were seen in the cervical spine AP when no instructions and no collar were used (28.3 mm). For cervical spine lateral movements, no collar but with instructions produced the greatest movement (18.5 mm). For the lumbar spine, the greatest movement was also recorded with no collar but with instructions (153.5 mm AP and 101.1 mm LAT).

When the data were disaggregated by gender similar findings were found for males and females, with application of a collar and no instructions leading to the smallest movements at the cervical and lumbar spine in both groups.

## Discussion

This is the first biomechanical analysis of different types of self-extrication published to date, reporting both cervical and lumbar movements as well as providing additional details of excursion and rotation. This is also the first study which allows direct comparison of the effect of instructions and cervical collars on spinal movement. The use of a collar and no instructions resulted in the smallest movement of the cervical and lumbar spine during self-extrication.

### Instructions

Commonly people remain in cars following MVC’s as a result of concerns about movement exacerbating potential spinal injury. Delivery of instructions would require the presence of trained personnel (rescue service or clinical) on scene or a telecommunications surrogate (e.g. via mobile telephone). If instructions are not beneficial, as suggested by this study, then this would potentially release clinical and operational personnel to other tasks and empower policy that encourages potential casualties to leave their car before the arrival of clinical or operational services.

The finding of increased spinal movement with instructions was unexpected. Dixon et al. utilised instructions for all of their self-extrications, which were also adopted for Haske’s single participant study [23, 25]. Engsberg et al. did not provide instructions to their participants [33]. Gabreli et al. compared the use of instructions provided in video and verbal explanatory format prior to the subjects (all young men less than 30 years of age) attempting self-extrication – they found that instructions reduced movement in the sagittal (AP) plane (other movements were not tested / analysed) at the cervical spine [24]. No previous studies have considered movement at the lumbar spine. Within our study we attempted to maximise external validity by using participants unfamiliar with extrication and using direct verbal instruction as would be delivered by a member of a rescue team at the scene of an incident.

We suggest that the smaller movements found when no instructions were given was a result of subjects finding their own ‘route’ to leave the vehicle, resulting in a more natural, comfortable extrication. This ‘naturalness’ perhaps explains the very narrow confidence interval found for results for no-collar and no instructions across all translation movements (Figs. 2, 3, 4 and 5). If this hypothesis is correct, we would expect the difference in movement between instructions and no instructions to be larger in a patient’s own vehicle, where familiarity and well-practiced egress could lead to smaller movements. We did not investigate the effect of variations in instructions but utilised the instructions previously produced by Dixon et al. – refinement of such instructions could lead to decreased spinal movement and is a consideration in planning further research in this area.

### Cervical collars

Cervical collars are carried on all FRS appliances in the UK. They are commonly applied to casualties whilst still in their car and remain in situ throughout extrication. If collars are not required in casualties suitable for self-extrication this would have significant implications for the time in their clinical course that casualties may be asked to attempt self-extrication. This could mean that some casualties could be asked to attempt self-extrication at initial call to the Emergency Services. Such a finding would also have significant implications for recommendations to bystander / buddy care at the scene of a motor vehicle collision. In our study there was a strong association between collar use and decreased cervical spinal movement (*p* < 0.001); this finding is in keeping with the intended purpose of such devices and is consistent with previous work [25, 33]. It is contrary to the findings of Dixon et al. who identified a small, mean increase in movement associated with collars when degrees of anterior–posterior, medial–lateral and rotational movement were combined [23]. The difference identified by Dixon was small, not present in all of the participants studied and the confidence intervals between the two groups overlapped. There has been increasing challenge to the routine use of cervical collars in prehospital care [22]. The purpose of a cervical collar is to minimise movement and as such stop an unstable fracture from causing secondary avoidable cord damage. A majority of the biomechanical analysis in this area uses healthy volunteers or cadavers and as a result it remains unclear that using a collar effectively reduces movement when an unstable cervical spine injury is present [34].

As might be expected, in our study the cervical collar did not consistently reduce movement at the lumbar spine.

### Movement in the context of spinal cord injury

Significant force is required to cause unstable spinal fracture or cord injury. Such forces would normally be associated with significant movement, movement that is likely to be maximal at the point of energy transfer. Despite the potential biomechanical implausibility of small additional movements causing further cord injury, extrication strategies and rescue services approach are focused on movement minimisation and the prevention of secondary injury [18].

### Limitations

This study has a number of limitations. By definition, our volunteers were healthy and without spinal pathology. They were not subjected to motor vehicle collisions, recent spinal trauma and did not have unstable (or other) spinal injuries. Our volunteers did not have distracting injuries, intoxication, confusion, pain-relief administered, or the psychological impact of a real MVC.

This limits application of our results to the significantly injured patient population. In real patients with spinal injuries, the movements may be larger in those with unstable injuries or reduced due to the pain and muscular spasm that frequently co-exists with an acute injury.

This study aimed to maximise external validity by utilising volunteers with no knowledge of the process of extrication, a mix of males and females and a range of weights, heights and BMI’s. There was no discernible association between each of these factors and spinal movement. In this context, variation of self-extrication technique by patient sex, age, weight, height or BMI cannot be recommended on the basis of this study, but could be considered in further research. The order in which participants progressed through the study arms was delivered to minimise learning, particularly in relation to the verbal instructions. Learning may, however, have occurred as the participants progressed through the study and this may affect the internal validity of the study. Likewise, the potential effect of participant fatigue on our results cannot be ruled out.

The study vehicle was the same for all volunteers and was not modified but was not one the participants were familiar with and it is possible that familiar vehicles would be associated with different extrication characteristics compared to our test vehicle. There may also be variation in results for vehicles with inherently different structural characteristics, for example, 4 × 4 type vehicles or low-riding sports vehicles.

### Interpretation in a clinical context

The majority of casualties involved in MVCs are uninjured or have only minor injuries it is this subgroup in which self-extrication is the preferred route of extrication and which has the most similarities to our healthy volunteers [11]. There are several potential advantages of self-extrication over tool extrication including decreased time, decreased resource utilisation and less risk to the patient and rescuer. Within the inherent limitations of this study, this work helps us to understand self-extrication in the context of spinal movement minimisation. When a patient is suitable for self-extrication (very few casualties with unstable injuries have occult injuries [22], instructions are unnecessary, could be counter-productive and should not be delivered. In services which use collars, these may be applied to facilitate extrication and then removed once the extrication is complete to minimise any potential complications. Further work is needed in this area to understand the movements associated with application of a collar to a patient in a car and the benefits and harms of collars in this patient group at the various stages of their patient journey.

Previous researchers have concluded that self-extrication is associated with smaller movements at the cervical spine than other methods of extrication, which normally involve being physically lifted from the vehicle by rescue service personnel on to a board or a scoop [23, 24]. Trapped casualties have an excess mortality, and many of the injuries they suffer are time critical [11]. As such, the benefits and harms of current extrication techniques need to be carefully considered in the context that in all likelihood the current approach is not achieving the intended therapeutic goals in terms of movement minimisation and are potentially contributing to excess morbidity and mortality.

### Future research

This should aim to answer the questions of which casualties should self-extricate, whether the principles identified here can be applied to other motor vehicles and the real-world resource, health economic and clinical benefits (or otherwise) of the adoption of self-extrication as the principle route of extrication for appropriate casualties following MVCs. Additional biomechanical studies should be designed to characterise the movement associated with in-car collar application and analysis of other commonly used extrication techniques, including those who cannot self-extricate.

Future research is needed to define which casualties may benefit from current movement minimisation techniques and furthermore engage with casualties and subject matter experts to identify a balanced solution to the problem of casualties trapped in vehicles following MVCs.

## Conclusion

In this study of healthy volunteers, self-extrication with no instructions but with a collar resulted in the smallest spinal movement of the four self-extrication approaches used. When a casualty is suitable for self-extrication, the instructions used in this study should not be used and a simple instruction to leave the vehicle delivered. In services which use collars, these may be applied to minimise spinal movement during extrication.

It is unlikely that the movement minimisation focus of current extrication techniques achieves its therapeutic goal and may contribute to the excess mortality of casualties who are trapped. The harms and benefits of current extrication strategies need careful consideration in this context.

## Data Availability

The datasets used and/or analysed during the current study are available from the corresponding author on reasonable request.
